# Molecular background of cadmium tolerance in Rht dwarf wheat mutant is related to a metabolic shift from proline and polyamine to phytochelatin synthesis

**DOI:** 10.1007/s11356-020-08661-z

**Published:** 2020-04-15

**Authors:** Gabriella Szalai, Judit Tajti, Kamirán Áron Hamow, Denyicska Ildikó, Radwan Khalil, Radomira Vanková, Petr Dobrev, Svetlana P. Misheva, Tibor Janda, Magda Pál

**Affiliations:** 1 Centre for Agricultural Research, 2462, Martonvásár, H-2462 Hungary; 2 Faculty of Science, Benha University, Benha, 13518 Egypt; 3 Institute of Experimental Botany of the Czech Academy of Sciences, 165 02 Prague, Czech Republic; 4 Institute of Plant Physiology and Genetics, Bulgarian Academy of Sciences, 1113 Sofia, Bulgaria

**Keywords:** ABA, PCs, PA, Proline, Rht, SA

## Abstract

Plant height is among the most important agronomic traits influencing crop yield. Wheat lines carrying *Rht* genes are important in plant breeding due to their both higher yield capacity and better tolerance to certain environmental stresses. However, the effects of dwarf-inducing genes on stress acclimation mechanisms are still poorly understood. Under the present conditions, cadmium stress induced different stress responses and defence mechanisms in the wild-type and dwarf mutant, and the mutant with the *Rht-B1c* allele exhibited higher tolerance. In the wild type after cadmium treatment, the abscisic acid synthesis increased in the leaves, which in turn might have induced the polyamine and proline metabolisms in the roots. However, in the mutant line, the slight increment in the leaf abscisic acid content accompanied by relatively high salicylic acid accumulation was not sufficient to induce such a great accumulation of proline and putrescine. Although changes in proline and polyamines, especially putrescine, showed similar patterns, the accumulation of these compounds was antagonistically related to the phytochelatin synthesis in the roots of the wild type after cadmium stress. In the dwarf genotype, a favourable metabolic shift from the synthesis of polyamine and proline to that of phytochelatin was responsible for the higher cadmium tolerance observed.

## Introduction

Cadmium is one of the major toxic metal pollutants, influencing and inhibiting several physiological and biochemical processes (Sanita di Toppi and Gabbrielli 1999). However, plants have evolved several defence mechanisms to minimise the damage caused by exposure to non-essential metal ions (DalCorso et al. 2008). The nitrate and sulphate assimilation pathways activated shortly after exposure to heavy metal play an important role in the increase in phytochelatin (PC) accumulation (Astolfi et al. 2004). These small molecule weight peptides are synthesised from glutathione in a reaction catalysed by phytochelatin synthase (PCS) and are capable of chelating metal ions in order to detoxify them via vacuolar compartmentalisation (Pál et al. 2006, 2018a). Other defence mechanisms involve the synthesis of protective compounds, such as proline or polyamines (PAs) (Balestrasse et al. 2005), and stress signalling molecules, such as salicylic acid (SA) (Kovács et al. 2014), abscisic acid (ABA) (Tran and Popova 2013) or gibberellins (GAs) (Zhu et al. 2012).

It has been revealed that the wheat dwarf mutant line *Rht-B1c* is more tolerant to drought and Cd stress than the tall wild-type line *Rht-B1a* (Kocheva et al. 2014a, b; Nenova et al. 2014; Dobrikova et al. 2017). The wild allele *–B1a* at locus *Rht-B1* encodes for DELLA proteins, which act as transcriptional repressors of the GA signalling pathway and retard plant growth (Achard and Genschik 2009). The mutant allele *–B1c* at the same locus (formerly designated as the *Rht3* gene) encodes for aberrant DELLA proteins that are unable to interact with the GA receptor, resulting in reduced GA responsiveness (Wen et al. 2013). The better stress tolerance of the *Rht-B1c* line has been related to alterations of the photosynthetic membranes, the increased capacity of the photosystem I cyclic electron transport, the accumulation of osmoprotectants and the higher activity of certain antioxidant enzymes (Dobrikova et al. 2017). However, the effects of wheat dwarfing genes, encoding modified DELLA proteins, on stress acclimation mechanisms are still poorly understood, as is the role of GAs under Cd stress (Asgher et al. 2014). Although Cd, Cu and Zn treatments have been reported to increase the level of GA_3_ in the roots of *Arabidopsis* plants, this increment was not related with the induction of glutathione or PC synthesis (Sofo et al. 2013). Thus, interactions between GA signalling and PC synthesis still need further research.

Apart from their direct protective effects, PAs also act as signalling molecules, with a role in the regulation of stress tolerance, and are involved both in direct interactions with a number of metabolic routes and in hormonal crosstalk (Pál et al. 2015). Transgenic *Arabidopsis* plants that constitutively overexpress the gene encoding arginine decarboxylase, which is responsible for the synthesis of the diamine PA putrescine (PUT), exhibited a reduction in both the amounts of GA_1_, GA_4_ and GA_9_, and in the transcript levels of *AtGA20ox1*, *AtGA3ox1* and *AtGA3ox3* (Alcázar et al. 2005), suggesting that PUT accumulation represses GA synthesis. Recent results showed that the *Rht-B1c* mutation responsible for the dwarf phenotype may also affect the PA metabolism in wheat plants (Pál et al. 2019).

Accordingly, the objective of the present study was to reveal the role of the mutant DELLA-encoding gene in Cd tolerance. Little information is available on the relationship between plant growth regulators, such as plant hormones or PAs, and PC synthesis under Cd stress. The responses of the dwarf mutant have not yet been compared with those of the wild type. Very few studies have been done on the relationship between GA and SA, a plant hormone playing a role in stress responses (Navarro et al. 2008; Alonso-Ramírez et al. 2009; Gallego-Giraldo et al. 2011). Thus, this study focused on elucidating the relationship between PC synthesis and changes in the PA metabolism and in SA and ABA synthesis after Cd exposure in plants carrying the wild allele *Rht-B1a* and in dwarf plants with the *Rht-B1c* allele.

## Materials and methods

### Plant materials, growth conditions and treatments

Near-isogenic lines of the wheat (*Triticum aestivum* L.) variety ‘April Bearded’: a dwarf line carrying the mutant allele *Rht-B1c* (formerly *Rht3*) and a tall counterpart, carrying the wild-type allele *Rht-B1a* (WT) were used in this study (Flintham et al. 1997). The improved Cd tolerance of this dwarf line compared with the wild type was manifested as better photosynthetic activity (Dobrikova et al. 2017).

After germination for 3 days at 26 °C in the dark, wheat seedlings (15/beaker) were grown in modified Hoagland solution in a Conviron GB-48 plant growth chamber (Controlled Environments Ltd., Winnipeg, Canada) according to as it was described by Pál et al. (2019). After 10 days of growth under normal conditions, half of the seedlings were treated with 50 μM Cd(NO_3_)_2_ in a hydroponic system for 7 days. Leaves and roots of the plants were collected from control and Cd-treated wheat plants for biochemical and gene expression studies on the 7th day of the Cd treatment.

### Determination of cadmium and proline contents and lipid peroxidation

The Cd content was determined using the inductively coupled plasma-atomic emission spectrometry method (ICP-AES, Jobin-Yvon Ultima 2 sequential instrument) (Anton et al. 2012). The proline content was measured according to Bates et al. (1973) as the absorbance at 520 nm using a spectrophotometer. The detection of lipid peroxidation was based on the determination of malondialdehyde (MDA) at 532 nm after subtracting the non-specific absorption at 600 nm, using an extinction coefficient of 155 mM^−1^ cm^−1^ (Thomas et al. 2004).

### Determination of phytochelatin content and phytochelatin synthase activity

The in vivo PC contents and in vitro PCS activity (PCS: EC:2.3.2.15) were analysed by HPLC according to Chen et al. (1997), as modified by Szalai et al. (2013). Root or leaf samples weighing 750 mg were ground with 750 μl of extraction buffer consisting of 50 mM Tris-HCl buffer, pH 8.0; 10 mM β-mercaptoethanol (βME); 1 mM phenylmethylsulfonyl fluoride; and 14% (*v*/*v*) glycerol. After centrifugation for 10 min at 10,000×*g*, the supernatant was used for the standard assay. The mixture for PCS contained 200 μl of the supernatant, 25 μl of 2.4 M Tris-HCl buffer (pH 8.0), 25 μl of 6 mM Cd(NO_3_)_2_, 25 μl of 120 mM βME and 25 μl of 120 mM GSH. After incubation for 60 min at 35 °C, the reaction was terminated by adding 30 μl of 50% sulfosalicylic acid followed by incubation on ice for 5 min. Sulfosalicylic acid was added to the same mixture without incubation (0 min) to determine the initial PC level (which also represents the in vivo PC level). PCs were analysed on a reverse phase column (Hypersil ODS, 100 × 2.1 mm, 5 μm, Thermo Scientific) with post-column derivatisation using Ellman reagent, in an Alliance 2690 system equipped with a UV W996 photodiode array detector (Waters, Milford, MA, USA) at 412 nm. The PCs were quantified using a glutathione calibration curve. The specific activity of PCS was calculated using PC produced during the 60-min incubation compared with the initial level and was expressed as nanomoles PC per minute per gram FW.

### Polyamine analysis

After homogenising samples with perchloric acid, the extract was centrifuged at 10,000*g* at 4 °C for 10 min and the supernatant was used for the pre-column derivatisation with dansyl chloride (Németh et al. 2002). PUT, spermidine (SPD) and spermine (SPM) were analysed together with 1,3-diaminopropane (DAP), the terminal catabolic product of SPD and SPM, by HPLC on a reverse phase Kinetex column (C18, 100 × 2.1 mm, 5 μm, Phenomenex, Inc., CA, USA) using a W2690 separation module and a W474 scanning fluorescence detector with excitation at 340 nm and emission at 515 nm (Waters, Milford, MA, USA).

### Polyamine oxidase enzyme activity

The activity of polyamine oxidase (PAO, EC 1.5.3.3.) was estimated spectrophotometrically as described by Takács et al. (2016). Enzyme activity was expressed as nanomoles of Δ^1^-pyrroline per minute per gram FW using an extinction coefficient of 1.86 × 10^3^ mol^−1^ cm^−1^.

### Extraction of salicylic acid and abscisic acid: analytical procedure

Both extraction and ultra-performance liquid chromatography with tandem mass spectrometry (UPLC-MS/MS) were carried out according to Vrhovsek et al. (2012) with slight modifications, described in detail by Pál et al. (2019). Briefly, after extraction with methanol:water (2:1) to a final sample ratio of 100 mg FW/ml, the UPLC-MS/MS analysis was performed on a Waters Acquity I class UPLC system coupled to a Waters Xevo TQ-XS (Milford, MA, USA) equipped with a UniSpray ion source (US) operated in timed MRM mode, where argon was used as a collision gas. Separation was achieved on a Waters Acquity HSS T3 column (1.8 μm, 100 mm × 2.1 mm), maintained at 40 °C. Water and acetonitrile gradients were used and both mobile phases contained 0.1 *v*/*v* % formic acid. Data processing was performed using Waters MassLynx 4.2 and TargetLynx software.

### Gene expression analysis

After extracting total RNA from plant tissues using TRI Reagent®, the samples were treated with DNase I and cleaned with a Direct-zol™ RNA MiniPrep Kit (Zymo Research, Irvine, CA, USA). The quality and integrity of RNA was monitored using agarose gel, and the samples were quantified with a Nanodrop 2000 Spectrophotometer (Thermo Fisher Scientific Inc., Wilmington, MA, USA). One thousand nanograms of total RNA was reverse-transcribed using M-MLV Reverse Transcriptase (Promega Corporation, Madison, WI, USA) and oligo(dT)18 (Thermo Fisher Scientific). A CFX96 Touch™ Real-Time PCR Detection System (Bio-Rad, Hercules, CA, USA) was used for the quantitative real-time PCR reaction, which involved 1 μl of 2-fold diluted cDNA, gene-specific primers (Table 1), housekeeping primer (*Ta2291* encoding for an ADP-ribosylation factor described by Paolacci et al. (2009)) and the PCRBIO SyGreen Mix (PCR Biosystems, London, UK). The relative gene expression values were determined with the 2^−ΔΔCt^ method (Livak and Schmittgen 2001). All the reactions were performed in triplicate using three biological and three technical repetitions.

**Table 1:** Gene-specific and housekeeping primers

Gene name	Primer sequences (5′ → 3′)		Reference
*Ta2291* (encoding ADP-ribosylation factor)	Forward	GCTCTCCAACAACATTGCCAAC	Paolacci et al. 2009
	Reverse	GCTTCTGCCTGTCACATACGC	
*TaNCED*	Forward	CCTCGAAGCCCAGCACTAAT	Gallé et al. 2013
	Reverse	GAGAGCGAGAGGTCCAATGG	
*TaP5CS1*	Forward	AGGCTGGGTATGAGAGTGC	Pál et al. 2018b
	Reverse	TAAGGCATCAGGTCGGGAC	
*TaPCS1*	Forward	CCTTCAAGCAGACTGGGACT	Tajti et al. 2018
	Reverse	GAGAAGCGTCAATGGAACCC	
*TaOAT*	Forward	TGATGATCGCTCGGCTTTACA	Pál et al. 2018b
	Reverse	CAGTAGCACCCATTGTTGCAG	
*TaPAO*	Forward	GCTCAAAATCAGCCAATTCCA	Xiong et al. 2017
	Reverse	TTCGCCATTTGTTGAGCTCT	
*TaICS*	Forward	TTCAGCTCCACCAAACCAACCA	Kovács et al. 2014
	Reverse	GGTTTGCCCACTGAAGAAGCG	

### Statistical analysis

Data are presented for the most representative repetition of the three independent biological experiments. The results are the means of at least five replicates, and the data were statistically evaluated using the standard deviation and Student’s *t* test methods.

## Results

### Effect of cadmium treatment on the biomass parameters, cadmium content and lipid peroxidation

The biomass parameters (Table 2) showed that 7-day treatment of 50 μM Cd only induced significant changes in the roots, where a decrease was observed in the length and weight in both the WT and Rht3 genotypes. The decrease in shoot length was not significant, but was more pronounced in Cd-treated wild genotypes (shoot length was 84.3% of the control for WT and 93.25% for Rht3).

**Table 2:** Effects of 7-day 50-μM Cd treatment on the biomass parameters of wheat genotypes (wild: WT and dwarf genotypes: Rht3). Data represent mean values ± SD. Different letters indicate significant differences at *P* ≤ 0.05 level

		Shoot		Root	
	Treatments	WT	Rht3	WT	Rht3
Length (cm)	Control	38.72 ± 3.29 b	17.44 ± 2.25 a	25.5 ± 0.71 b	21.5 ± 0.7 a
	50 μM Cd	35 ± 3.1 b	16.15 ± 2.75 a	21.5 ± 1.12 a	20.5 ± 0.8 a
Weight (g/plant)	Control	0.69 ± 0.14 b	0.38 ± 0.09 a	0.82 ± 0.24 c	0.43 ± 0.01 b
	50 μM Cd	0.65 ± 0.12 b	0.35 ± 0.11 a	0.42 ± 0.14 b	0.23 ± 0.04 a

Table 3 shows that Cd treatment resulted in high Cd accumulation in the roots of both genotypes. Cd was also translocated into the leaves. The increase in MDA content indicated that the Cd exposure caused oxidative stress, especially in the leaves. A significantly higher level of lipid peroxidation was detected in WT than in the Rht3 dwarf genotype. The results also indicated that the higher tolerance of the mutant line was not due to limited Cd absorption or translocation, as higher leaf Cd content was measured in dwarf plants than of the WT (Table 3).

**Table 3:** Effects of 7-day 50-μM Cd treatment on the cadmium content and the level of lipid peroxidation (malondialdehyde [MDA] level) in the leaves and roots of wheat genotypes (wild: WT and dwarf genotypes: Rht3). Data represent mean values ± SD. Different letters indicate significant differences at *P* ≤ 0.05 level

		Leaves		Roots	
	Treatments	WT	Rht3	WT	Rht3
Cd content (μg g^−1^ DW)	Control	—	—	—	—
	50 μM Cd	48.5 ± 4.3 a	114 ± 11.8 b	2394 ± 164 b	1976 ± 153 a
MDA (nmol g^−1^ FW)	Control	4.1 ± 0.7 a	4.5 ± 0.7 a	2.3 ± 0.2 a	2.4 ± 0.4 ab
	50 μM Cd	7.7 ± 0.5 c	6.1 ± 0.2 b	3 ± 0.4 b	3 ± 0.3 b

### Changes in proline synthesis after cadmium treatment

**Fig. 1:**
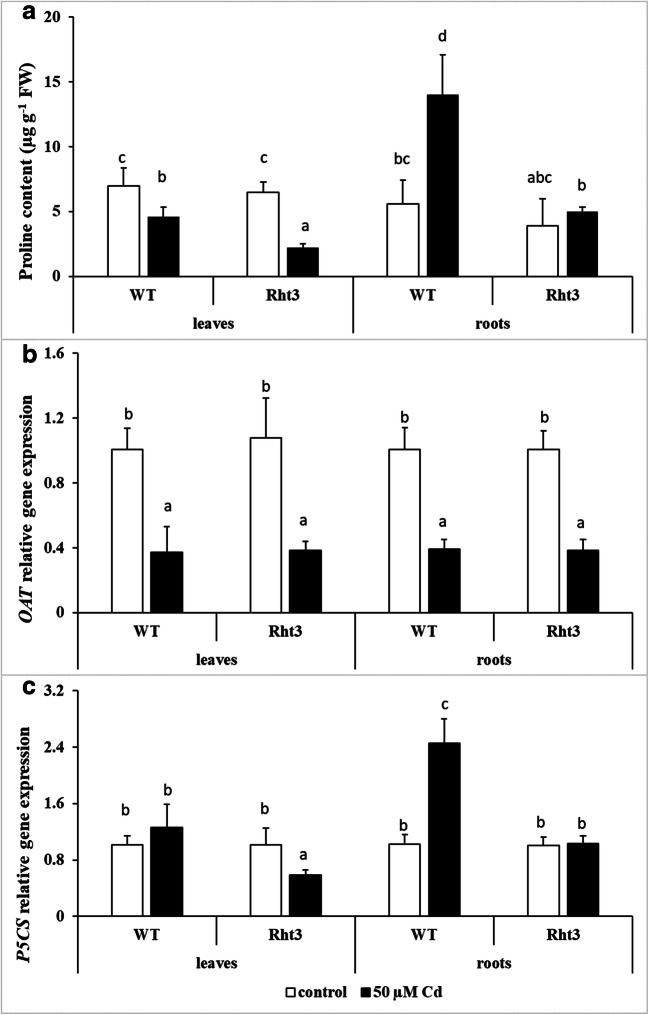
Effects of 7-day 50-μM Cd treatment on the proline content (**a**), and on the gene expression levels of ornithine aminotransferase (*OAT*: **b**) and Δ^1^-pyrroline-5-carboxylate synthase (*P5CS*: **c**) in the leaves and roots of wheat genotypes (wild: WT and dwarf: Rht3). Data represent mean values ± SD. Different letters indicate significant differences at the *P* ≤ 0.05 level

Cd stress reduced the amount of proline in the leaves of both genotypes, but to a greater extent in the Rht3 plants (Fig. 1a). In contrast, in the roots, high proline accumulation was observed only in the case of the WT. These changes only partially correlated with the expression levels of the *OAT* and *P5CS* genes (Fig. 1b, c). The transcript level of *OAT* decreased in the leaves and roots of both genotypes after Cd treatment, while that of *P5CS* only decreased in the leaves of Cd-treated Rht3 plants and increased in the roots of WT. These results suggested that *OAT* expression affected the proline content in the leaves, while the *P5CS* expression pattern correlated with proline levels in the roots, and only partly in the leaves.

### Phytochelatin content and phytochelatin synthase activity after cadmium treatment

**Fig. 2:**
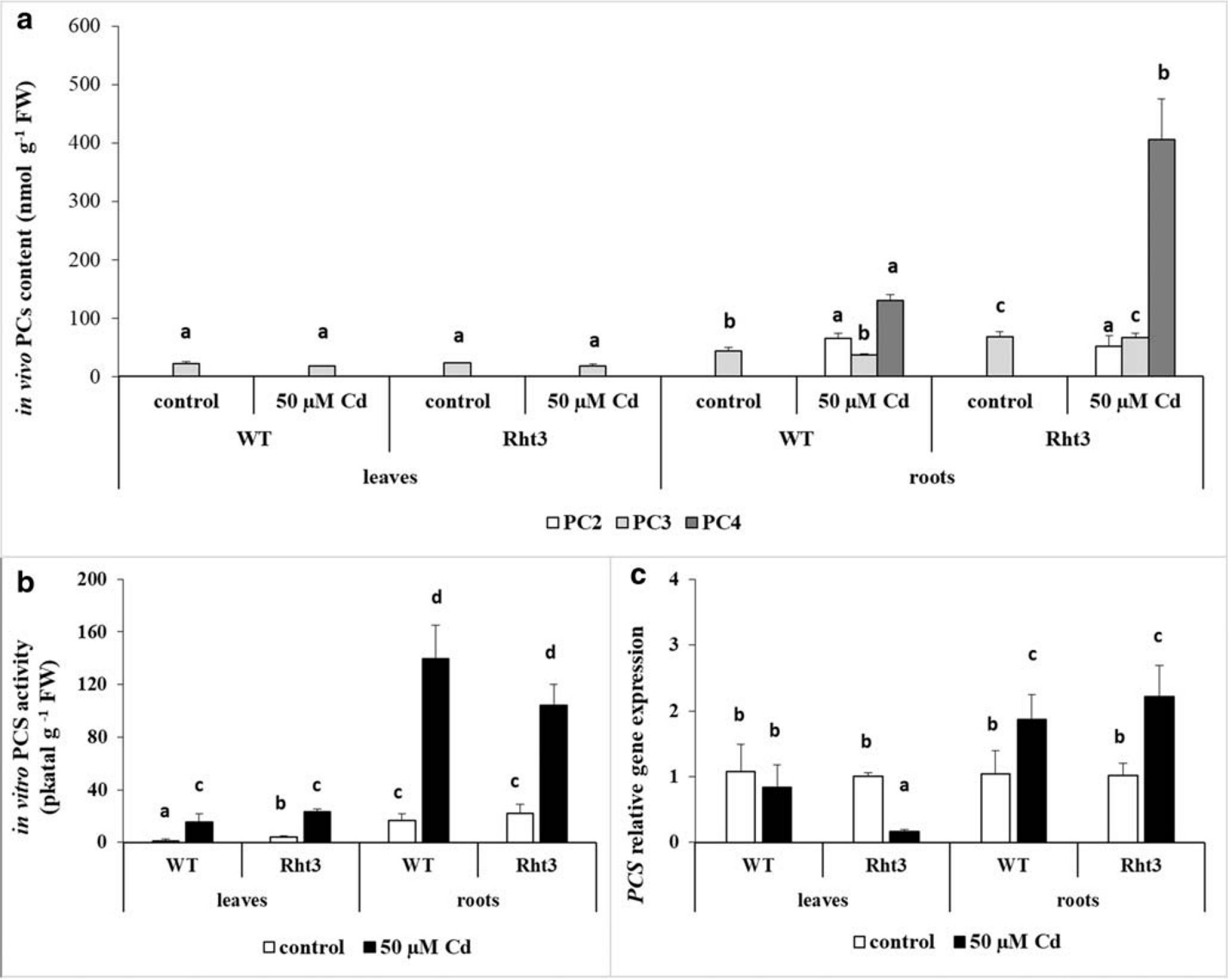
Effects of 7-day 50-μM Cd treatment on the in vivo phytochelatin (PC) content (**a**), in vitro phytochelatin synthase (PCS) activity (**b**) and gene expression level of phytochelatin synthase (*PCS*: **c**) in the leaves and roots of wheat genotypes (wild: WT and dwarf: Rht3). Data represent mean values ± SD. Different letters indicate significant differences between the PC_2_, PC_3_ and PC_4_ contents, the PCS activity and *PCS* gene expression level at the *P* ≤ 0.05 level

Only phytochelatin 3 (PC_3_) was detected in the leaves in vivo (Fig. 2a), and its amount was not influenced by Cd treatment. However, 7 days of 50 μM Cd treatment induced the synthesis of PCs in the roots. PCs with *n* = 2–4 could be detected in Cd-treated, wild-type and mutant genotypes (Fig. 2a). The highest accumulation was found for PC_4_ in Rht3 plants. The in vitro PCS activity was induced after Cd treatment, especially in the roots, but no profound difference was observed between the two genotypes (Fig. 2b). The *PCS* expression also increased in the roots of both genotypes (Fig. 2c), but it was downregulated in the leaves of Cd-treated Rht3 plants, probably due to the negative feedback regulation caused by the accumulation of PC_4_.

### Changes in the polyamine metabolism after cadmium treatment

**Fig. 3:**
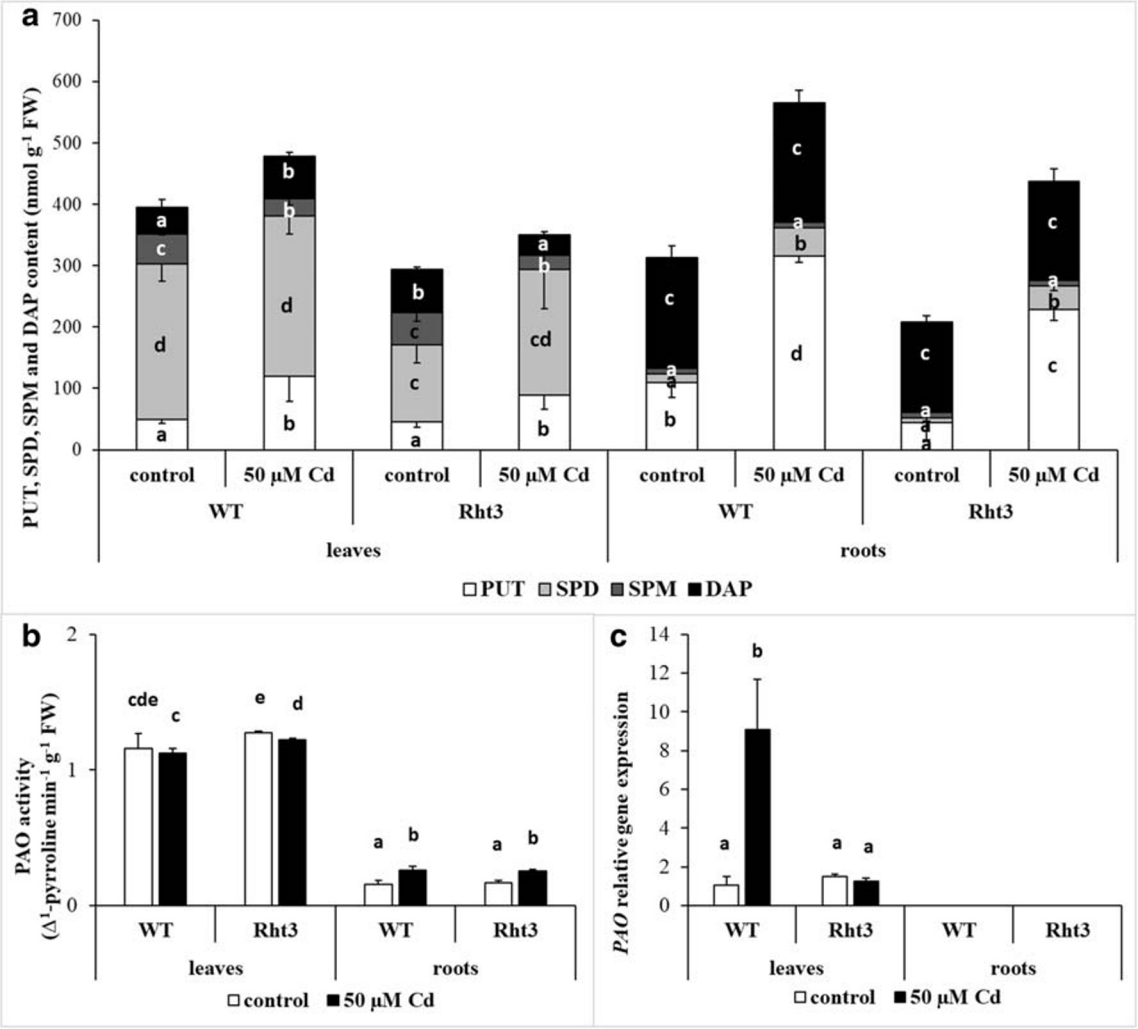
Effects of 7-day 50-μM Cd treatment on the polyamine content (PUT putrescine, SPD spermidine and SPM spermine) (**a**), polyamine oxidase (PAO) activity (**b**) and gene expression level of polyamine oxidase (*PAO*: **c**) in the leaves and roots of wheat genotypes (wild: WT and dwarf: Rht3). Data represent mean values ± SD. Different letters indicate significant differences at the *P* ≤ 0.05 level

Pronounced differences in the initial PA contents were observed in the two genotypes tested. The mutant exhibited a lower level of SPD in the leaves, but a higher content of DAP. In the roots, the mutant contained less PUT than the WT plants (Fig. 3a). Cd stress increased the level of PUT in the leaves and roots of both genotypes, with the highest increment in the case of Cd-treated WT roots. A significant increase in the SPD levels was only observed after Cd stress in the roots, while the quantity of SPM decreased in the leaves of both genotypes. The level of DAP, the catabolic product of SPD and SPM, exhibited slight, but significant increase in the leaves of WT, while decreasing in the Rht3 genotype (Fig. 3a). Parallel with changes in SPD, SPM and DAP, the activity of the PAO enzyme, catalysing the terminal catabolism of SPD and SPM, showed no pronounced change. The PAO activity in the leaves was high even under control conditions and was not influenced by Cd treatment. In the roots, the initial low activity rose only slightly after Cd stress (Fig. 3b). The expression of the *PAO* gene was only detected in the leaves, where Cd treatment increased it dramatically in WT, but not in Rht3 (Fig. 3c).

### Effect of cadmium stress on plant hormones, SA and ABA

**Fig. 4:**
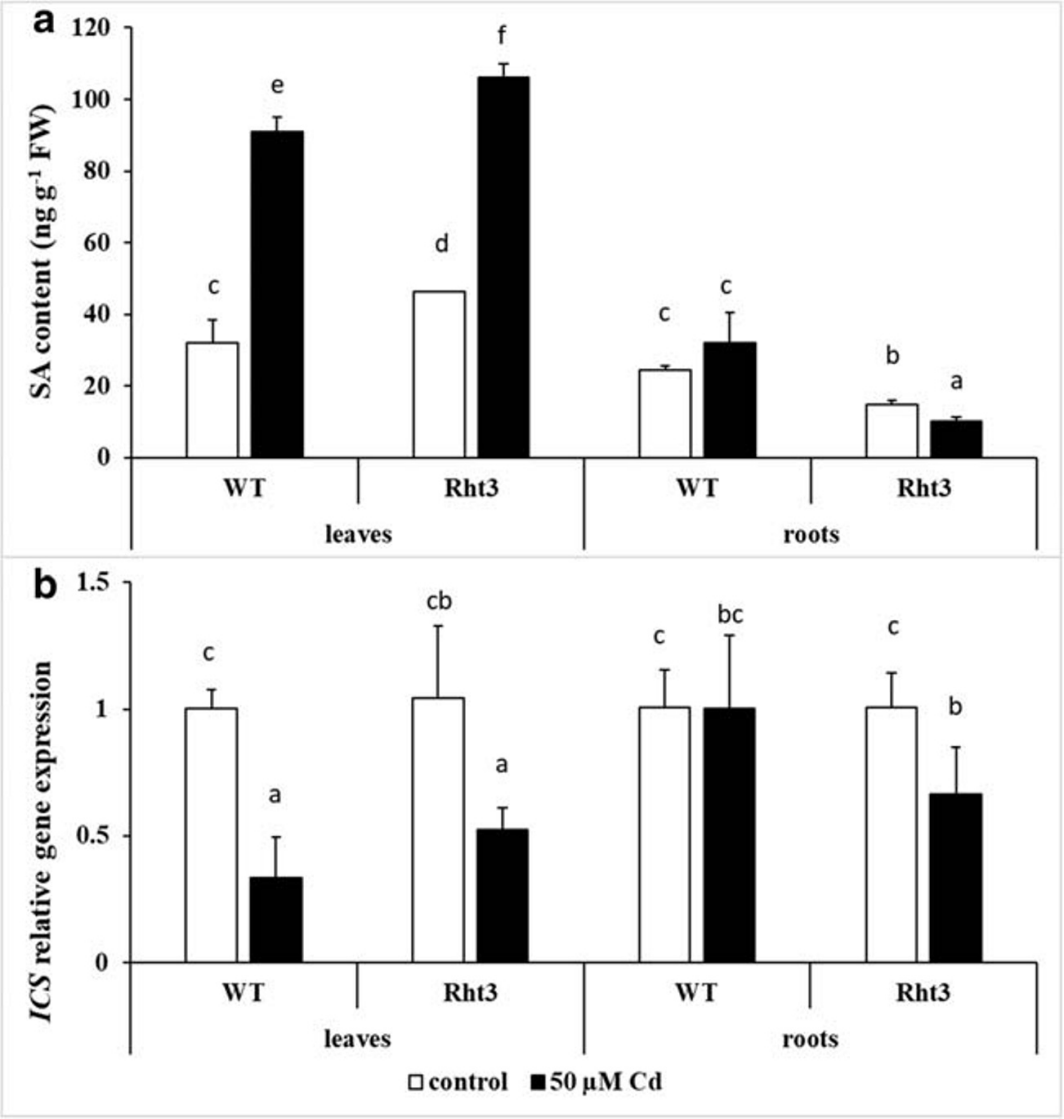
Effects of 7-day 50-μM Cd treatment on the salicylic acid (SA) content (**a**) and on the gene expression level of isochorismate synthase (*ICS*) (**b**) in the leaves and roots of wheat genotypes (wild: WT and dwarf: Rht3). Data represent mean values ± SD. Different letters indicate significant differences at the *P* ≤ 0.05 level

Cd stress resulted in SA accumulation in the leaves of both genotypes, especially in Rht3, where the basal SA level was also slightly higher (Fig. 4a). Interestingly, the amount of SA did not change in the roots of WT, but decreased slightly in Rht3. The expression of *ICS*, a gene encoding one of the SA biosynthesis enzymes, was downregulated in the leaves of Cd-treated plants (Fig. 4b), showed a trend parallel with that of the SA level in the roots.

**Fig. 5:**
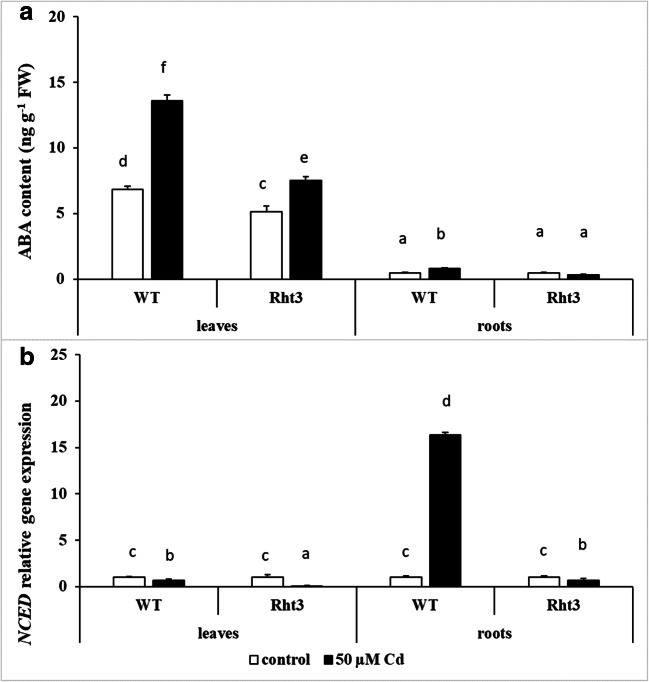
Effects of 7-day 50-μM Cd treatment on the abscisic acid (ABA) content (**a**) and on the gene expression level of the 9-cis-epoxycarotenoid dioxygenase gene (*NCED*) (**b**) in the leaves and roots of wheat genotypes (wild: WT and dwarf: Rht3). Data represent mean values ± SD. Different letters indicate significant differences at the *P* ≤ 0.05 level

Cd treatment also increased the ABA content in the leaves of both genotypes, but to a greater extent in WT (Fig. 5a). The ABA level was much lower in the roots than in the leaves, only increasing slightly after Cd treatment in WT. Although the transcription of *NCED*, the gene encoding the key enzyme in ABA synthesis, decreased slightly in the leaves of Cd-treated plants, its expression showed a dramatic increase in the roots of Cd-treated WT (Fig. 5b).

## Discussion

It was recently demonstrated that *Rht-B1c* mutation (encoding GA-insensitive DELLA proteins) is responsible for the greater tolerance of wheat plants to Cd stress, which was manifested as better photosynthetic activity (Dobrikova et al. 2017). In the present study, further investigations were performed at the molecular level to provide more information on the background of the different levels of Cd tolerance in the wild-type and mutant lines.

The results confirmed those of Dobrikova et al. (2017), as the Cd-induced root length inhibition was relatively lower observed for the Rht3 genotypes, while the MDA data indicated that the Rht3 line, with repressed GA signalling, had better tolerance of Cd. Changes in the synthesis of proline, PA, PC, ABA and SA were also evaluated to highlight differences between WT and the more Cd-tolerant dwarf genotypes under Cd stress conditions.

### Relationship between proline and PC synthesis

When Cd enters the cytosol, it activates the synthesis of several metal-binding compounds, such as PC, proline and PA (Chmielowska-Bąk et al. 2014). Cd-induced increases in proline content were reported for a number of plant species (Leskó et al. 2000; Al Khateeb and Al-Qwasemeh 2014). Exogenous proline was also found to protect membrane integrity and reduce ROS production in *Solanum nigrum* exposed to Cd stress (Xu et al. 2009). Under the present conditions, Cd stress decreased the proline content in the leaves of both genotypes, but increased it in the roots of WT. In a similar manner, proline accumulation was found to be closely associated with a reduction in root growth in Cd-treated rice roots (Chen and Kao 1995). Proline can be synthesised directly from glutamate in a reaction catalysed by Δ^1^-pyrroline-5-carboxylate synthetase (P5CS) or from ornithine by ornithine aminotransferase (OAT). However, it is not always clear which pathway is involved in increased proline biosynthesis directly from glutamate by P5CS or from ornithine by OAT under stress conditions (Majumdar et al. 2016). For example, recently, it was found that 1-day ABA treatment induced the gene expression of both enzymes, but 1-day PUT treatment only the *P5CS1*, while osmotic stress after 5 days did not induced any changes in the transcript level either of these gene, despite the fact that proline accumulation occurred. In addition, it was also revealed that the synthesis pathway of PA and proline is regulated partly independently, despite the common precursor, glutamate (Pál et al. 2018b). Furthermore, it should be also taken into consideration that not only the syntheses of PA and proline are linked but also the PA catabolism has also been shown to be closely related to proline accumulation (Aziz et al. 1998). The present results revealed that differences in the root proline content between the wild and mutant genotypes induced by Cd could be attributed to the altered expression level of *P5CS*, and not that of *OAT.* In addition, higher proline accumulation was parallel with the highest total PA level in the roots of the Cd-treated WT plants. However, differences in the proline content alone could not explain the better tolerance of Rht3 to Cd.

PC synthesis has been demonstrated to be a major metal detoxification mechanism in plants. Basal *PCS* expression was detected even without heavy metal exposure, but it is strongly activated by metal ions (Zitka et al. 2011; Pál et al. 2018a). In wheat, Cd tolerance could be explained by the fast and profound induction of PC production (Kovács et al. 2014). In the present study, Cd also induced the formation of PCs in the roots of the two genotypes tested, the greatest accumulation being observed for PC_4_, which was much higher in the Rht3 mutant. However, no differences could be detected in the in vitro PCS activity or *PCS* gene expression, which may suggest that the only limiting factor is the availability of the precursor for PC synthesis (cysteine) during competing with PA synthesis. Since PCs play an important role in the detoxification of heavy metals, they may be responsible for the higher Cd tolerance observed for the dwarf line.

Earlier findings highlighted a possible link between proline synthesis and PC accumulation (Siripornadulsil et al. 2002). Investigations on the green alga *Chlamydomonas reinhardtii*, a *P5CS* overexpressor, suggested that the increased proline accumulation during Cd stress helped to reduce oxidative damage and contributed to a more reducing cellular environment, which may then have led to a higher glutathione level, facilitating PC synthesis (Siripornadulsil et al. 2002). Although proline has been reported to chelate metals (Sharma et al. 1998), the metal-chelating ability of tomato plants was correlated with the gene expression level of *PCS*, but not with that of *P5CS* or proline content under Cd stress (Kısa 2019). In addition, despite the indirect metabolic connection between the proline and PC synthesis, the Cd concentration-dependent induction of the *PCS* expression was accompanied with lower increment in the *P5CS* transcript level, if any, and a decrease in the proline content (Kısa 2019). Similarly, lower *P5CS1* transcription level and lower proline accumulation were found under the present conditions in Rht3 roots, where the highest total PCs level was measured.

### Relationship between PA and PC synthesis

According to the above described connection between proline and PA synthesis, in addition the suggested indirect relationship between the synthesis of proline and PCs, in order to reveal more connections, the PA metabolism was analysed to elucidate the cause of the different levels of Cd tolerance in the wild-type and mutant lines at the molecular level. The synthesis of PAs is related not only to proline (PAs and proline have glutamate as a common precursor) but also in the case of SPD and SPM, with PCs (cysteine is a precursor for the synthesis of both glutathione and S-adenosylmethionine, which is the aminopropyl donor for the synthesis of SPD and SPM), so there seems to be an important metabolic relationship between these protective compounds. Increased contents of PA, especially PUT, and proline have been reported in soybean after Cd stress, but these did not prevent Cd-induced changes in nitrogen assimilation pathways and the Cd stress response (Balestrasse et al. 2005). In addition, it has been demonstrated in rice that during Cd stress, PAs may have a substantial influence on PC synthesis, as exogenous PUT inhibited both the activity and the gene expression level of PCS (Pál et al. 2017). SPD is synthesised from PUT through the addition of an aminopropyl moiety donated by decarboxylated S-adenosylmethionine and catalysed by SPD synthase, while adding an additional aminopropyl moiety to SPD produces SPM with the help of SPM synthase (Liu et al. 2015). SPD and SPM can also be metabolised by FAD-containing polyamine oxidases (PAOs), which can be divided into two groups, the first of which catalyses the terminal catabolism of SPD and SPM, and producing DAP in the apoplast, while the second is responsible for peroxisomal back-conversion, from SPM, via SPD, to PUT (Cona et al. 2006; Moschou et al. 2008). According to these, the PA cycle involves the synthesis and the back-conversion of the PAs. In the present experiment, *PAO* expression could only be detected in the leaves and was only induced in WT after Cd stress. The PAO enzyme activity in the leaves was much higher than in the roots of both genotypes and was not substantially influenced by Cd stress. Parallel with the changes in leaf DAP, there was no change in the SPD content in WT, but the SPM level decreased, while in Rht3 plants, the SPD level increased and SPM decreased, despite the fact that the PUT content increased in the leaves of both genotypes. These differences suggest that the terminal catabolism of higher PAs was induced in WT, while the interconversion of SPM to SPD and decreased terminal catabolism were responsible for the increment of leaf SPD content in Rht3. These results suggest that the observed cellular PA level was rather due to the controlled PA transport into the apoplast, than the activation of apoplastic PAO activity in the WT under Cd stress. Similarly, in tobacco, increased exodus of newly produced PA into apoplasts was reported under stress conditions (Yoda et al. 2003). This is also in accordance with the changes in the *PAO* expression, which shows the increasing need for PAO enzyme, and terminal catabolism only in the Cd-treated WT plants, where the highest PA accumulation was found. In the roots, the SPD content increased in both wild-type and mutant plants, in accordance with the increased PUT level, indicating that PA synthesis was induced after Cd stress. However, the fact that the initial leaf SPD content in WT was double, that in Rht3 plants may be related to the lower inducibility of PC synthesis in the roots under Cd stress, indicating an antagonistic relationship between PCs and higher PAs, because of the common precursor.

PAs were also reported to act as metal chelators (Lomozik et al. 2005). The present results, which showed opposite patterns for PC and PA dynamics, suggest that the tested genotypes used different strategies under cadmium stress conditions, with WT accumulating more proline and PAs, especially PUT, while the dwarf increased PC_4_ production.

### Relationship between proline, PCs, PAs and plant hormones

The interaction between the compounds analysed (proline, PCs and PAs) and plant hormones (Pál et al. 2015, 2017, 2018a, b) makes the picture even more complex. SA synthesis starts from chorismate, as a precursor, which after, the synthesis pathway is branching to two separated pathways. SA can be synthesised through the phenylalanine ammonia-lyase (PAL) pathway from phenylalanine via benzoic acid, while on the isochorismate pathway, the isochorismate synthsase (ICS) catalyses isochorismate formation from chorismate. Cd treatment caused concentration-dependent SA accumulation in maize (Pál et al. 2005), and in wheat, SA accumulation was parallel with the increased activity of PAL (Kovács et al. 2014). Under the present conditions, SA accumulation was observed in the leaves of both genotypes, but the *ICS* expression decreased in the leaves after Cd stress. The latter was probably due to the inhibitory feedback effect of the SA accumulation in the chloroplasts, where ICS catalyse the synthesis of SA.

Although numerous studies have been reported on the positive effect of SA during Cd stress, very few experiments have focused on its effect on PC accumulation. Exogenous SA influenced the PC content and PCS activity in the leaves of maize plants (Gondor et al. 2016), while in barley, it alleviated Cd toxicity, though the protection was not related to the transcription level of *PCS* (Metwally et al. 2003). In another study, soaking seeds in SA before Cd treatment reduced the Cd-induced oxidative stress and influenced the PC composition in maize plants, while at higher Cd concentrations greater PC accumulation was found compared with plants treated only with Cd (Szalai et al. 2013).

Cd treatment was also reported to increase the ABA content in potato, in parallel with the upregulation of the PC synthesis, which latter was also induced after ABA treatment (Stroiński et al. 2010). Although studies involving the inhibition of ABA synthesis indicated that ABA is required in Cd signal transduction, ABA pre-treatment before Cd stress had negative effect on PC synthesis (Stroiński et al. 2013). In the present study, Cd induced the accumulation of both SA and ABA in the leaves of both wild-type and mutant genotypes, but there were some differences. The accumulation of SA was slightly higher after Cd exposure in the leaves of the dwarf line, while that of ABA was lower. This is not surprising, as DELLAs are positively affected by ABA (Zentella et al. 2007; Xu et al. 2014), so their stabilisation may have a negative feedback effect. More and more studies suggest an antagonistic relationship between SA and ABA signalling (de Torres-Zabala et al. 2007; Meguro and Sato 2015; Manohar et al. 2017), which may explain the lower level of ABA in the dwarf genotype under the present conditions.

These differences in SA and ABA dynamics were only observed in the leaves. In the roots, the SA content tended to decrease while that of ABA did not change in Cd-treated Rht3 plants, but in the WT, Cd exposure greatly increased the expression of *NCED*. However, in contrast to *ICS*, *NCED* gene is not feedback-regulated by the end product, ABA. Indeed, increased ABA content can increase *NCED* expression. According to these, an important regulatory mechanism of the actual ABA level is it hydroxylation and degradation (Liu et al. 2016).

The higher root *NCED* level was parallel with a substantial ABA accumulation in the leaves, accompanied by the intense induction of *PAO*, also in the leaves, and may also be responsible for the increased *P5CS* expression and proline content in the roots of Cd-treated WT. These results agree with finding that ABA influences the synthesis of proline both at the transcriptional level, via the upregulation of *P5CS* expression, and at the post-transcriptional level, by stabilising transcripts of *P5CS* (Hare et al. 1999), and also confirm the fact that ABA treatment enhances the activity of PAO and the expression of the gene coding for PAO in maize (Xue et al. 2009). In addition, higher ABA content may increase the expression level of the genes involved in PA metabolism (Alcázar et al. 2006). Similarly, ABA application and accumulation resulted in proline accumulation and increased gene expression of *P5CS1*, *OAT* and *PAO* in wheat (Pál et al. 2018b). Our data indicated that there is no direct correlation between changes in PCs and SA or ABA contents in the roots. However, it was found in *Sedium alfredii* that too high ABA accumulation weakens or even reverses the ABA-induced Cd stress responsive gene expression (Lu et al. 2020), which suggest that although ABA has regulatory role in Cd-induced stress responses, higher accumulation of it can shift the metabolic processes into less beneficial direction.

## Conclusion

This is the first study to characterize the relationships between proline, PCs and PAs on the one hand, and the plant hormones SA and ABA on the other hand under Cd stress in a GA-insensitive mutant wheat line. The results confirmed that PCs play an important role in the heavy metal detoxification and that this may underlie the higher Cd tolerance observed in the dwarf line. The results also indicated that Cd stress induced ABA synthesis in the wild type, which in turn enhanced the proline and PA metabolism. In contrast, the mutant line only exhibited a slight increment in leaf ABA content, which was not sufficient to induce high proline or PUT accumulation in either the leaves or roots, but this was favourable for the synthesis of PCs.

## Abbreviations

ABA: Abscisic acid
DAP: 1,3-Diaminopropane
GAs: Gibberellins
ICS: Isochorismate synthase
MDA: Malondialdehyde
NCED: 9-cis-Epoxycarotenoid dioxygenase
OAT: Ornithine aminotransferase
P5CS: Δ^1^-Pyrroline-5-carboxylate synthase
PC: Phytochelatin
PCS: Phytochelatin synthase
PUT: Putrescine
SA: Salicylic acid
SPD: Spermidine
SPM: Spermine
